# *In Vitro* Dermal Safety Assessment of Silver Nanowires after Acute Exposure: Tissue *vs*. Cell Models

**DOI:** 10.3390/nano8040232

**Published:** 2018-04-11

**Authors:** Sylvia G. Lehmann, Benjamin Gilbert, Thierry GG Maffeis, Alexei Grichine, Isabelle Pignot-Paintrand, Simon Clavaguera, Walid Rachidi, Michel Seve, Laurent Charlet

**Affiliations:** 1 Univ. Grenoble Alpes, Univ. Savoie Mont Blanc, CNRS, IRD, IFSTTAR, ISTerre, 38000 Grenoble, France; Charlet38@gmail.com; 2 Energy Geoscience Division, Lawrence Berkeley National Laboratory, Berkeley, CA 94720, USA; bgilbert@lbl.gov; 3 Centre for Nanohealth, College of Engineering, Swansea University, Swansea SA1 8EN, UK; t.g.g.maffeis@swansea.ac.uk; 4 INSERM, UGA, Institute for Advanced Biosciences, F-38000 Grenoble, France; alexei.grichine@univ-grenoble-alpes.fr; 5 Univ. Grenoble Alpes, CNRS, Grenoble INP, F-38000 Grenoble, France; isabelle.paintrand@grenoble-inp.fr; 6 Univ. Grenoble Alpes, F-38000 Grenoble, France; simon.clavaguera@cea.fr; 7 CEA, LITEN, DTNM, NanoSafety Platform, F-38054 Grenoble, France; 8 Univ. Grenoble Alpes, INAC, SyMMES, F-38000 Grenoble, France; walid.rachidi@cea.fr; 9 CEA, INAC, SyMMES, F-38054 Grenoble, France; 10 Univ. Grenoble Alpes, LBFA et BEeSy, PROMETHEE Proteomic Platform, F-38000 Grenoble, France; michel.seve@univ-grenoble-alpes.fr; 11 INSERM, U1055, PROMETHEE Proteomic Platform, F-38000 Grenoble, France; 12 CHU Grenoble Alpes, Institut de Biologie et de Pathologie, PROMETHEE Proteomic Platform, F-38000 Grenoble, France

**Keywords:** silver nanowires, cytotoxicity, skin irritation *in vitro*, primary keratinocytes, 3D reconstructed epidermis model

## Abstract

Silver nanowires (AgNW) are attractive materials that are anticipated to be incorporated into numerous consumer products such as textiles, touchscreen display, and medical devices that could be in direct contact with skin. There are very few studies on the cellular toxicity of AgNW and no studies that have specifically evaluated the potential toxicity from dermal exposure. To address this question, we investigated the dermal toxicity after acute exposure of polymer-coated AgNW with two sizes using two models, human primary keratinocytes and human reconstructed epidermis. In keratinocytes, AgNW are rapidly and massively internalized inside cells leading to dose-dependent cytotoxicity that was not due to Ag^+^ release. Analysing our data with different dose metrics, we propose that the number of NW is the most appropriate dose-metric for studies of AgNW toxicity. In reconstructed epidermis, the results of a standard *in vitro* skin irritation assay classified AgNW as non-irritant to skin and we found no evidence of penetration into the deeper layer of the epidermis. The findings show that healthy and intact epidermis provides an effective barrier for AgNW, although the study does not address potential transport through follicles or injured skin. The combined cell and tissue model approach used here is likely to provide an important methodology for assessing the risks for skin exposure to AgNW from consumer products.

## 1. Introduction

In the past 10 years, silver nanowires (AgNW) have emerged as an attractive material for a range of applications including transparent, flexible, conductive electrodes and thin films. With competitive properties to indium tin oxide (ITO), such as high optical transparency and low sheet resistance, but at lower cost and with resistance to flexing, they are predicted to replace ITO in solar cells, touch screen, liquid and flexible displays [1,2,3]. 

Although many studies have investigated the toxicity of silver nanoparticles (AgNP), finding a significant role for the release of Ag^+^ ions and oxidative stress induction [4,5], few studies have been dedicated to AgNW. Key studies using models for respiratory exposure have demonstrated that AgNW confirm at least partially to the fibre pathogenicity paradigm, with a length threshold for pulmonary inflammation and toxicity in rodents and frustrated phagocytosis by macrophages *in vivo* and *in vitro* [6,7,8,9,10]. Additional *in vitro* studies with alveolar epithelial cells, non-immune cell models for respiratory exposure, have observed variable measures of toxicity. In A549 cells, exposure to AgNW induces some cytotoxicity in one study [11], but not in another one at low doses [12]. In other cell lines, such as TT1 and ATII, no toxicity was observed at the concentration tested although an increased inflammatory response was observed [13,14]. 

Although respiratory exposure is certainly relevant to occupational health, dermal exposure may be a more important risk for consumers due to the development of touchscreen displays [15,16], wearable textiles [17,18,19], and medical devices incorporating AgNW [20,21]. Anticipating the development of AgNW-enabled touchscreens, Verma *et al.* exposed a range of cell lines (epithelial, endothelial, gastric, and phagocytic) to 3–6 µm long AgNWs, in suspension or embedded in a transparent electrode, finding for the suspension AgNW a low level of cytotoxicity at a relatively low maximum dose (5 µg/mL) varying depending on cell type and increasing with AgNW doses and time of incubation [22]. These studies point to risks for AgNW exposure to numerous cell lines but have not provided a clear understanding of the conditions leading to adverse outcomes and are just an initial step for evaluating the potential hazard for the consumer. 

Here we describe an investigation into the dermal route of exposure to AgNW. We performed paired AgNW uptake and cytotoxicity studies in human primary keratinocytes (HPK) cells and in reconstructed human epidermis (RHE) model (epiCS). Indeed, HPK are the major cell component of the epidermis and RHE is an alternative model that presents the three-dimensional architecture of skin tissue, including the outer protective barrier. RHE models are used for dermal exposure to cosmetics and environmental irritants and pollutants with approved OECD guidelines like the *in vitro* skin irritation assay [23]. Such an approach has been used to determine the extent of penetration and the toxicity of several nanomaterials [24,25], but not yet for AgNW.

## 2. Materials and Methods 

### 2.1. Silver Nanowires Characterization

Short and long polyvinylpyrrolidone (PVP) coated AgNW suspensions (S-AgNW and L-AgNW) were purchased from nanoComposix (San Diego, CA, USA) and were stored in lightproof bottles in an anaerobic chamber. Both solutions of AgNW were imaged by SEM and the length and diameter observed were in agreement with manufacturer specifications and with previous observations [26] (Table S1). 

AgNW were supplied into water at 1 mg/mL suspension. They were diluted into complete keratinocytes culture medium, then agitated gently for 1 min and they were well dispersed without aggregation, as observed throughout this study by light microscopy (data not shown). AgNW settling rates were assessed by measuring a series of optical absorption spectra (Ocean Optics, Largo, FL, USA) of unstirred 4-mL suspensions of 1 µg/mL S-AgNW and L-AgNW in keratinocyte culture media (KSFM; Life Tech., Villebon Sur Yvette, France) at different times. AgNW present a peak of absorption at 380 nm, the decrease of absorbance at 380 nm over the time reflect then AgNW settling. Measurements were performed in closed 1-cm path-length quartz cuvettes. 

The calculation of AgNW concentration expressed as the number of AgNW/mL was made using the following formula:

N = C/(π × r^2^ × l × d)

where N = number of wires/mL; C = concentration (g/mL); π = Pi; r = radius; l = length; d = density. The calculated volume and surface area/single NW is indicated in Table S1.

### 2.2. Ionic Silver Release into Media

Ag^+^ release into complete keratinocytes culture medium was quantified by adding AgNWs to culture medium and measuring the dissolved silver concentration at different intervals with ICP-MS (Perkin-Elmer Elan DRC II) as previously described [26].

### 2.3. Cell Culture

Normal human keratinocytes were isolated from skin tissue obtained after breast plastic surgery, from healthy Caucasian donors (32 to 34 year old) with their informed consent (Department of “Chirurgie Plastique et Maxillo-faciale”, CHU Grenoble, La Tronche, France). Keratinocytes were isolated from the skin explant as described previously [27] and cells from at least three different donors were used for each experiment. Human primary keratinocytes were then cultured in keratinocyte serum free medium (KSFM; Life Tech.) supplemented with 25 µg/mL Bovine Pituitary extract (BPE), 0.9 ng/mL recombinant human epithelial growth factor (EGF) and primocin (Life Tech., Villebon Sur Yvette, France) at 37 °C in a humidified atmosphere containing 5% CO_2_. For all experiments cells were used at passages 2 or 3 only.

Three dimensional human reconstructed epidermis epiCS (CellSystems, GmbH, Troisdorf, Germany) were maintained according to the suppliers’ instructions in 6-well plates at 37 °C, 5% CO_2,_ and 95% relative humidity with epiCS medium. 

### 2.4. Cell Viability Assays

Cytotoxicity was assessed using the 3-[4,5-dimethylthiazol-2-yl]-2,5-diphenyl tetrazolium bromide (MTT) assay (Sigma-Aldrich, St. Louis, MO, USA). 

Human Primary Keratinocyte (HPK) assays were performed as follows. Cells were seeded in 96-well plates in complete KSFM medium. After 24 h, cells were treated with concentrations of 1.56, 3.13, 6.25, 12.5, 25, and 50 µg/mL of L-AgNW, S-AgNW and AgNO_3_ as a control for silver ion toxicity. Silver nitrate (99.999% trace metal basis) was obtained from Sigma-Aldrich. After 24 h, cells were washed two times with phosphate buffered saline (PBS) and fresh KSFM medium was added supplemented with 0.5 mg/mL MTT. After 3 h of incubation at 37 °C, formazan crystals were dissolved with 100 µL Dimethyl sulfoxide (DMSO) and then transferred into new plates for absorbance reading at 560 nm using a microplate reader (Varioskan^®^ Flash, Thermo Scientific, Villebon-sur-Yvette, France). Cell viability was expressed relatively of absorbance of negative control (non-treated cells). Data analyses were performed with Excel 2013 for Windows. In order to ensure that there was no interference between the MTT and AgNW, the same experiment was performed without cells and the NW were pelleted by centrifugation (15 min at 800 g) before the addition of MTT. The absorbance measured was similar to the background (data not shown). 

In order to determine if Ag^+^ ions released from the AgNW had any contribution to the observed loss of viability, we performed an ion-release experiment. After 24-h incubation with cells, cell supernatants were harvested, centrifuged (15 min at 800 g), and the supernatants were transferred onto other cells for another 24 h prior to a second MTT assay that was performed as described previously. 

### 2.5. In Vitro Skin Irritation Assay with epiCS RHE

Reconstructed Human Epidermis (RHE) assays were performed as follows. After 24 h of equilibration time, RHE were exposed topically to 50 µL of 0.6 mg/mL of S-AgNW, L-AgNW and the equivalent concentration of Ag^+^ as AgNO_3_. A positive control with 3% Sodium dodecyl sulfate (SDS) was also performed. All dilutions were performed in sterile water and the suspensions fully covered the RHE surface. The same amount of water was added topically to the control non-treated samples. The treatment was applied as recommended in the OECD Test Guideline 439 [23] (*in vitro* skin irritation assay) for 20 min, then the RHE was washed 20 times in PBS and incubated 42 h. We also performed a longer exposition time, when no washes step was performed and incubation lasted 42 h. At the end of the 42 h, RHE were rinsed with PBS and MTT assay was performed according to the manufacturer’s instructions. Absorbance measurement and analysis of the data were done as previously stated. 

### 2.6. Electron Microscopy Analysis (SEM and TEM)

HPK were grown on glass coverslips and incubated with 1.56 µg/mL of AgNW for 24 h. Then, HPK were rinsed with Hanks Balanced Salt Solution, fixed in 4% paraformaldehyde prepared in a 0.1-M sodium cacodylate buffer at pH 7.2 (Sigma-Aldrich, St. Louis, MO, USA), dehydrated in 5 min baths of increasing concentrations of ethanol (70; 80; 85; 90; 95, and 100%) and then the samples were allowed to air dry at RT. Whole cells were observed with a Hitachi S4800 scanning electron microscope (SEM) (Centre for Nanohealth, College of engineering, Swansea, UK). Most samples were imaged without electrical charging artefacts but a few cases were coated with a Cr coating of 5 nm. 

Some HPK samples treated for 72 h were fixed in 2.5% glutaraldehyde in a 0.1-M sodium cacodylate buffer at pH 7.2, post-fixed in 1% osmium tetroxide and 1.5% potassium hexacyannoferrate(II) trihydrate, dehydrated with 30 min baths of increasing concentrations of ethanol (70; 95 and 3 times in 100%) and embedded in Epon resin (Delta Microscopy). Sections were cut (250 nm) with an ultramicrotome (Leica (Wetzlar, Germany), NanoID, CEA) and observed with a FEG SEM ZEISS GeminiSEM 500 (CMTC, France) using a backscatter detector. The contrast of images was inverted with Image J (NIH).

RHE samples that were exposed to AgNW for 42 h were rinsed with PBS, fixed in 2.5% glutaraldehyde in a 0.1-M sodium cacodylate buffer at pH 7.2, post-fixed with 1% osmium tetroxide for 1 h, counterstained with 0.5% uranyl acetate at pH 4 overnight, then dehydrated in graded concentrations of ethanol and embedded in Epon resin (Delta Microscopy). Ultra-thin and semi-fine sections (60 nm and 400 nm, respectively) with an ultramicrotome (Ultracut Leica) and observed with a JEOL 1200 EX transmission electron microscope (TEM) operated at 80 kV (Grenoble Institut des Neurosciences, France). Semi-thin sections were stained with toluidine blue and visualized with a phase contrast microscope to assess RHE morphology.

### 2.7. Confocal Microscopy

HPK were grown on Lab-Tek™ chamber slides and incubated for 24 h with 1.56 µg/mL of AgNW. DNA was stained by the addition of Hoechst 33342 (1 µg/mL in PBS, Sigma) and images were acquired with a LSM710 confocal microscope (IAB, Grenoble, France). Hoechst fluorescence was excited with 405 nm laser and detected in 420–480 nm interval in confocal descanned mode with a pinhole diameter of 1 Airy unit. The AgNW were detected in confocal reflected light mode. For time lapse analysis, HPK were incubated immediately before imaging with 1.56 µg/mL of AgNW. Three-dimensional (3D) stack images were acquired using a 40X/1.2 water immersion objective with Zen software (Carl Zeiss, Oberkochen, Germany). The top and bottom points of the cells were defined, and 29 z-stacks with a step size of 0.5 µm per time point were acquired each 2 min for 8 h with a LSM 510 Carl Zeiss confocal microscope (IAB, Grenoble, France). Image analysis was performed with Image J (NIH).

### 2.8. Cytoviva Microscopy

HPK were grown on glass coverslips and exposed for 72 h to 1.56 µg/mL of AgNW. After incubation, the cells were rinsed with PBS, fixed in 4% paraformaldehyde and stained with Hoechst 33342. Dark-field micrographs and hyperspectral images were acquired with a CytoViva hyperspectral microscopy system (CEA, NanoSafety Platform, Grenoble, France). The hyperspectral data were converted to text files using ENVI software and analysed in the IgorPro software using the GG Macros routines developed for spectromicroscopy. The lineshapes of all visible-to-near-infrared (V-NIR) spectra dominated the output of the lamp and the precise alignment of the condenser, making quantitative spectroscopy very difficult. However, by subtracting a spectrum from a nearby bright Ag-free biological structure, V-NIR spectra of AgNW and AgNR could be obtained. 

## 3. Results and Discussion

### 3.1. AgNW Characterization

**Figure 1:**
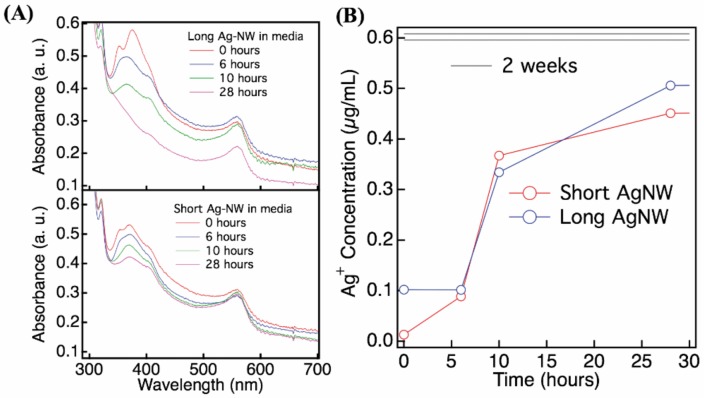
Physicochemical behavior of AgNW in keratinocyte culture medium (KSFM). Kinetics of short and long AgNWs settling (**A**) and dissolution (**B**) following incubation in KSFM medium measured by UV-vis (**A**) and ICP-MS (**B**). (**A**) Series of optical absorption spectra acquired from unstirred 50 µg/mL AgNW suspensions in KSFM. Drop in absorbance at 380 nm reflects AgNW settling. (**B**) Ag^+^ dissolution of a 50 µg/mL AgNW suspension over the time.

The two sizes of commercial PVP-coated AgNW used in this study came from the same supplier as prior studies and as described in the methods section. They possessed similar dimensions and minor nanorods (AgNR) contamination as described previously [9,26]. The average dimensions were approximately 2 µm × 40 nm (length × diameter) for the short samples (S-AgNW) and 20 µm × 50 nm for the long sample (L-AgNW) (Table S1). AgNW diluted into complete keratinocytes culture medium were well dispersed without aggregation, as observed throughout this study by light microscopy (data not shown). We followed AgNW settling in culture medium by measuring the decrease of absorbance at 380 nm (Figure 1A). L-AgNW settled faster than S-AgNW. Although the spectrophotometric data cannot easily be converted to settling rates, it is likely that the L-AgNW settled completely within 24 h in the well-plates used for toxicity assays. The release of Ag^+^ into cell-free media was measured for 50 µg/mL AgNW dilution by ICP-MS over the time (Figure 1B). After 28 h incubation, Ag^+^ concentration reached 0.4–0.5 µg/mL for the short- and long-NW respectively. Ag^+^ dissolution was measured also after 2 weeks of incubation and the concentration was 0.6 µg/mL for both types of AgNW.

### 3.2. Human Primary Keratinocytes 

#### 3.2.1. Acute Cytotoxicity

**Figure 2:**
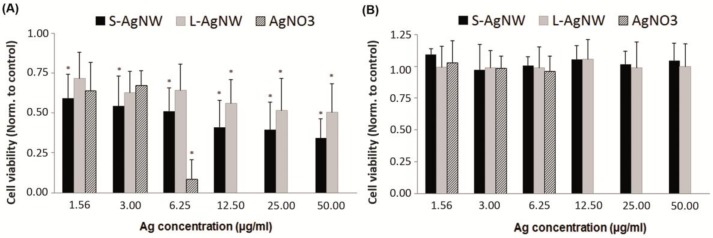
(**A**) Viability of primary human keratinocytes cells following 24 h exposure with short, long AgNWs and AgNO_3_ (represented each individually as bars from left to right respectively) determined by MTT assay. (**B**) Ion release control experiment. After 24 h incubation, cell medium containing AgNW or AgNO_3_ were collected and centrifuged. Then the supernatants were collected and transferred onto other cells for 24 h prior to the MTT assay. Cell viability data is presented as normalized to the non-treated control cells (*n* = 3) +SD; Significant differences between non-treated and treated cells are indicated by a * where *p* < 0.05.

The acute cytotoxicity of AgNW and Ag^+^ to human primary keratinocytes (HPK) was assessed using the MTT assay. Both types of AgNW induced moderate toxicity (Figure 2A) with a shallow dose response curve showing 35% and 50% viability after 24 h at the highest concentration tested (50 µg/mL) for the S- and L-AgNW, respectively. Much higher cytotoxicity was observed for Ag^+^ with a sharper dose response, zero cell viability at 12.5 µg/mL and a calculated LD50 of 4 µg/mL. Similar cytotoxicity was observed with the neutral red uptake assay (data not shown). 

In order to investigate the possible contribution of Ag^+^ release in the cell medium to the toxicity of AgNW, we harvested the cell supernatant after 24 h of exposure, centrifuged it, and added it to a fresh cell sample to perform an MTT assay after the 24-h incubation. All supernatants from AgNW exposure showed no toxicity at all (Figure 2B), consistent with the low measured Ag^+^ release, approximately 1% w/w (Figure 2B), and showing that the cytotoxicity observed after AgNW treatment is not due to Ag^+^ release in medium. Similar results were reported with A549 cells after 48 h of exposure [11] although Ag^+^ release in RPMI 1640 medium supplemented with 10% FBS was higher than observed here for the keratinocytes medium that does not contain serum. Verma *et al*. also performed this ion release control experiment and did not observe any toxicity of the AgNW supernatant in A549 cells cultivated in Ham’s F12 medium containing 10% FBS [22]. 

**Figure 3:**
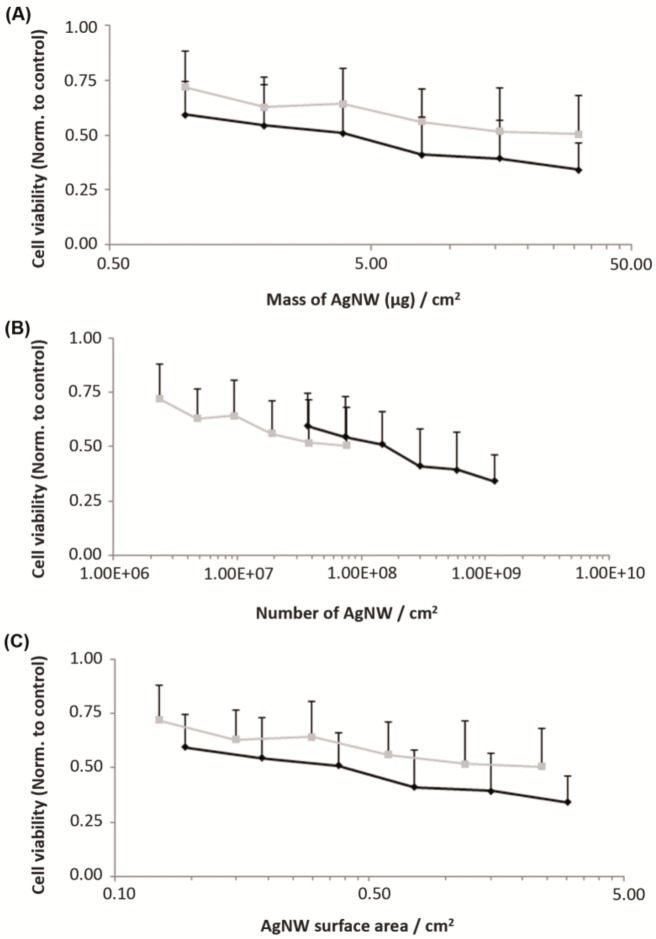
Viability of primary human keratinocytes cells determined by MTT assay following 24 h treatment with short and long AgNWs (black and grey line respectively). Cell viability data is presented as normalized to the non-treated control cells (*n* = 3) + SD and represented using different dose metrics: mass of AgNW (**A**), number of NW (**B**), and NW surface area (**C**) relative to the cell growth surface area expressed in cm^2^.

The supernatant from the AgNO_3_ exposures exhibited lower-toxicity responses in this second experiment than in the first one (Figure 3B). For example, while only ~10% viability was obtained in the first assay at 6.25 µg/mL Ag^+^, the supernatant from this sample gave 100% viability. This is consistent with a portion of the added Ag^+^ being removed from the media by sorption or uptake. 

Several reports [28,29] have suggested that mass concentration may not be the most appropriate dose metric for nanotoxicology studies. We then expressed our results using other metrics and in Figure 3 we compare cell viability relatively to mass of NW (Figure 3A), particle number (Figure 3B) and surface area (Figure 3C). We observed an almost linear dose-response trend across both data sets with particle number (Figure 3B). Thus our data on AgNW support the conclusions of previous studies [30,31] suggesting that particle number is the most relevant dose metric for fibre like nanomaterials. Also, S and L-AgNW induced similar toxicity, in agreement with prior work in which 1.5 and 8 µm AgNW induce a similar toxicity on A549 cells [11].

#### 3.2.2. Scanning Electron Microscopy 

**Figure 4:**
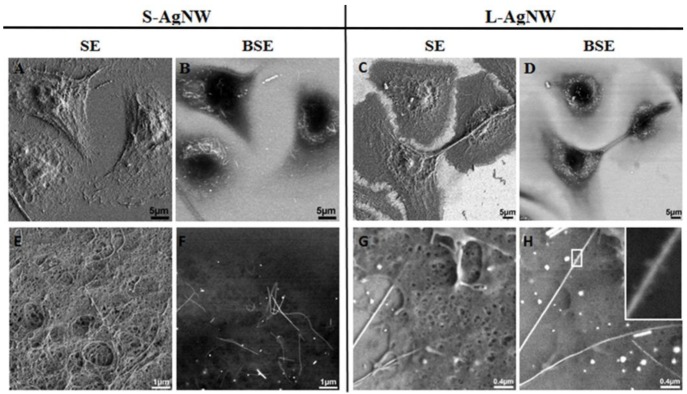
SEM images showing the uptake, the cellular distribution and changes of short (Left panel: A,B,E,F) and long (right panel: C,D,G,H) AgNWs in human primary keratinocytes cells after 24 h of exposure to 1.5 µg/mL AgNW. Images acquired at low (**A**–**D**) and high (**E**–**H**) magnification. SEM images were acquired using the secondary electron (SE; Left panel) and backscattered electron (BSE; Right panel) detector respectively. Scale bars represent 5 µm (**A**–**D**), 1 µm (**E**,**F**) and 0.4 µm (**G**,**H**) respectively. Changes in the morphology of AgNWs internalized inside cells is demonstrated (**E**,**H**) with bending (**F**) and a rough surface of internalized AgNW compared to their pristine surface outside cells (**G**,**H**). Insert in (**H**) show magnification of the box area and the modified AgNW surface.

SEM with secondary-electron (SE) detection provided images of the surface morphology of the whole HPK fixed cells while imaging with backscattered-electron detection (BSE) revealed both AgNW outside and inside cells with high contrast [7]. Using the two detectors, SEM analysis of whole HPK cells revealed efficient internalization of AgNW after 24 and 72 h of exposure. For example, HPK cells exposed to a low dose of AgNW for 24 h showed many examples of complete AgNW internalization (Figure 4A–D) as well as partially internalized AgNW (Figure S1). No AgNW-like structures were present in any control non-treated cell (Figure S2). 

**Figure 5:**
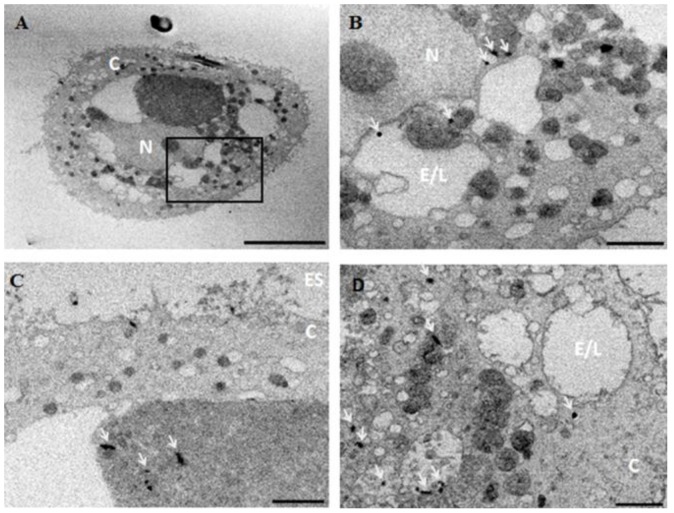
SEM images of cross sections of keratinocytes exposed to long AgNW for 72 h (**A**–**D**). Image B shows a higher magnification of the box area in (**A**). Sections of AgNW (highlighted by a white arrow) are localized in the cell cytoplasm, either free inside the cytoplasm or inside some endocytic vesicles. N = Nucleus, C = Cytoplasm, E/L = endosome/lysosome vesicles, ES = extracellular space. Scale bar represent 10 µm (**A**) and 2 µm (**B**–**D**).

SEM images of cross sectioned HPK cells also observed AgNW throughout the cell body (Figure 5). These electron-dense dots or rods were not present in cells that were not exposed to AgNW (Figure S3). Some AgNW were clearly localized inside endocytic vesicles but other AgNW were apparently present inside the cytoplasm at no specific location. The latter could have entered through an endocytic pathway and reached the cytoplasm through lysosome membrane damage, or may have pierced directly the cell membrane as demonstrated for some carbon nanotubes [32]. Internalized AgNW were never present inside the nuclear compartment, even after 72 h of exposure, in accord with a previous study of AgNW [14]. These results contrast with silver nanoparticles that were identified inside the nucleus of IMR-90 and U251 human cell lines [33], although the silver nanoparticles had a smaller diameter (6–20 nm) than the AgNW. 

Internalized S- and L-AgNW exhibited significant morphological alteration, including bending and breaking, not observed in AgNW that settled on the substrate without cell interactions (Figure 4F). The surfaces of AgNW that were internalized inside cells also showed an increased roughness (Figure 4H), similar to prior SEM images of AgNW located in the hemolymph of AgNW exposed *daphnia* [26]. Using high angle annular dark field scanning transmission electron microscopy (HAADF-STEM) at high magnification, Chen *et al.* demonstrated that sulphidization reactions leading to the formation of silver sulphide (Ag_2_S) occurred close to and on the surface of PVP-coated AgNW within human alveolar epithelial (TT1) cells [14]. It is likely that the surface modifications observed here represent similar oxidative sulphidation and reprecipitation reactions. 

#### 3.2.3. Confocal Fluorescence and Reflectance Microscopy

**Figure 6:**
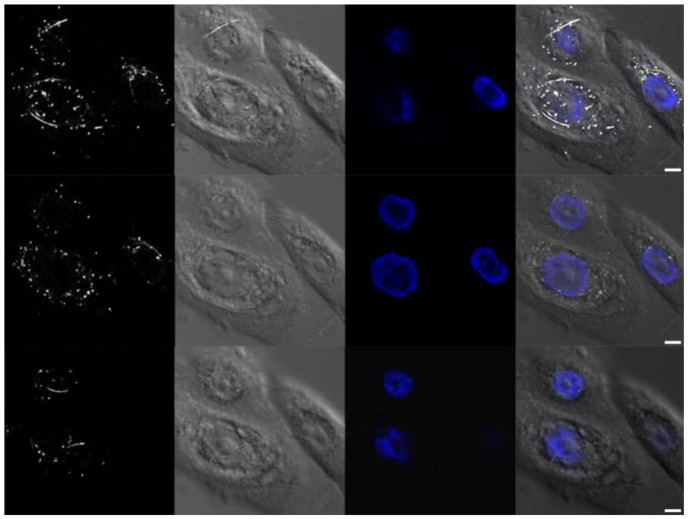
Confocal images showing the uptake and the cellular distribution of long AgNWs in human primary keratinocytes cells after 24 h of exposure to 1.5 µg/mL of L-AgNW. Scale bars represent 5 µm. Images were acquired in different mode (from left to right images): reflectance; transmittance; fluorescence in order to visualize respectively the NW; the cell and the nucleus that was stained with Hoechst. Image on the right is the merge of the three images. Images are shown at three different focal plans in order to visualize different depth in the cells.

Confocal microscopy of HPK cells that were fixed and stained with Hoechst following 24-h L-AgNW exposure revealed a high density of AgNW and AgNR throughout the cytoplasm (Figure 6 and Movie S1). Longer, unbroken AgNW were clearly wrapped around the cell nucleus which neither contained any detectable AgNW nor AgNR. Time-lapse confocal microscopy of live cells captured an apparent internalization event that occurred during a 2 min interval, 1 h 26 min. after the addition of L-AgNW to cells (Movie S2). This result shows that the kinetic of AgNW internalization is fast although the mechanism remains unclear. 

#### 3.2.4. Hyperspectral Microscopy

**Figure 7:**
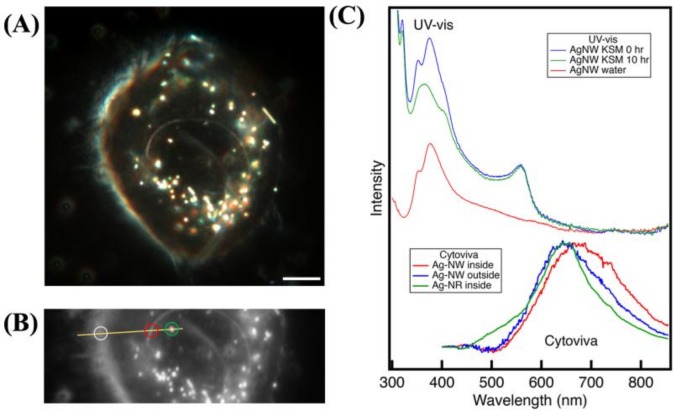
Hyperspectral analysis of AgNW and nanorods (AgNR) after 24 h of exposure in human primary keratinocytes (HPK). (**A**) Dark-field image of single HPK cell. (**B**) Region of HPK cell analyzed by visible-near infrared (V-NIR) hyperspectral microscopy. Circles indicate locations from where V-NIR spectra were extracted: white = reference spectrum; red = internal AgNW; green = internal AgNR. External AgNWs are outside this field of view (**C**) Processed V-NIR spectra compared with UV-vis absorbance from AgNW in HPK media.

Visible to near-infrared (V-NIR) spectra obtained from AgNW internalized by HPK cells consistently exhibited a red shift relative to AgNW that settled on the substrate (Figure 7). Quantification and interpretation of these spectral changes is challenging, particularly because the wavelength range of analysis does not overlap with the strong surface Plasmon resonance region of the optical excitation spectra. Moreover, the V-NIR response of Ag can be altered by several processes. For example, Pratsinis *et al.* used hyperspectral microscopy to locate AgNP internalized by murine macrophages (RAW 264.7 cells, ATCC) [34]. They observed a red-shift in some internalized objects attributed to aggregation. Leclerc and Wilkinson studied toxicity of AgNP and AgNO_3_ to *C. reinhardtii* [35]. They observed nanoparticulate Ag within algal cells following exposure to either AgNP or silver ion that exhibited a distinct spectral profile relative to external AgNP. This was interpreted as being due to secondary precipitation of Ag (S) following Ag^+^ diffusion into the cell. Because the SEM data are consistent with silver sulphidisation reactions, and because no internal aggregation of NW is observed, we interpret the spectral changes to be caused by surface chemical change. 

### 3.3. Reconstructed Human Epidermis

#### 3.3.1. Skin Irritation Assay and Histology

**Figure 8:**
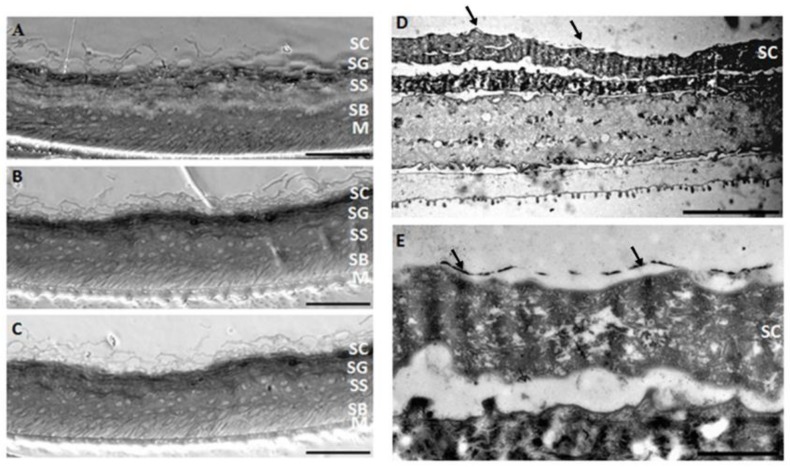
Phase contrast microscope (**A**–**C**) and TEM images (**D**,**E**) of reconstructed human epidermis (RHE) sections. No difference in RHE morphology was observed between non-treated (**A**) and s-AgNW (**B**) and L-AgNW (**C**) exposed RHE for 42 h. No AgNW was observed in the sections of RHE analysed (**D**,**E**), although the presence of AgNW lying at the surface of the stratum corneum or in the upper layer of the SC (**E**) is suspected (indicated by black arrow). The different layers of the skin are indicated. SC = stratum corneum; SG = stratum granulosum; SS = stratum spinosum; SB = stratum basale; M = membrane. The scale bar represent 20 µm (**A**–**C**); 10 µm (**D**) and 2 µm (**E**).

An *in vitro* skin irritation assay was performed with epiCS reconstructed human epidermis (RHE) models as recommended in the OECD test guideline 439 [23]. Any decrease in viability was observed after 20 min of contact with AgNW followed by extensive PBS washing and 42 h recovery (data not shown). This assay was also performed with 42 h of contact and, as described in Table 1, although the positive control (3% SDS) induced a drastic decrease of RHE viability, S-AgNW and Ag^+^ treatment generated a weak but significant decrease in viability (96.6% and 91.9% of viability respectively). According to these results, AgNW could then be classified as non-irritant to skin because the viability was higher than 50%. Moreover, RHE treated with AgNW showed a very similar histology to control non-treated samples (Figure 8A–C). 

A survey of the literature identified other nanomaterial toxicants that did not induce skin irritation using the OECD test guideline 439 and RHE models. For example, MWCNT at a dose of 10 mg/insert [25] and zinc oxide nanoparticles at a concentration 0.5 mg/mL [24] caused no decrease in RHE viability, even though the LD50 for the latter was 50 µg/mL in HaCat cell line. This difference in sensitivity between 2D cell culture and 3D RHE model is consistent with the efficient barrier protection of the reconstructed epidermis. In a recent study [36], silver nanoparticles with different sizes (10–100 nm) and with different coating (citrate, PVP or silica) as well as silver nitrate similarly did not induce skin irritation. In this study, the exposure time was 1 h followed by 42 h recovery and silver concentration was 1 mg/mL. 

#### 3.3.2. Transmission Electron Microscopy

We looked for evidence of cellular internalization of AgNW inside the epidermis model using TEM analysis of RHE ultrafine sections. Only one section out of five analysed showed any object consistent with the AgNW, in this case a bent NW lying on the surface of the stratum corneum layer (Figure 8D,E). Supporting elemental composition analysis was not available but the measured average diameter was in concordance with AgNW characteristics. Absence of penetration into epidermis of silver nanoparticles was also observed in vivo in pig even after topical repetitive daily application for two weeks [37], although, focal inflammation and oedema was observed with increasing AgNP concentration. 

Our results show that compared to 2D cells in culture, which internalize extensively AgNW, the 3D RHE model showed no sign of penetration, which is consistent with the barrier function of the epidermis and the literature for many nanoparticles [38,39]. Although, few examples of NP penetration into the viable layer of intact epidermis or even the dermis have been reported [40,41]. There is also strong evidence that the extent of nanomaterial penetration into the skin changes, when the integrity of the skin barrier is altered by several factors such as UV exposure, flexing, hydration status, skin age, chemical or physical damage, and skin pathologies [28,42,43]. For example Mortensen *et al.* showed quantum dot nanoparticles penetration into the viable epidermis and dermis after exposure to UV simulating a sunburn [44]. Two studies found that even particles up to 1 µm in size can be taken up through intact skin to reach living cells under mechanical stress such as flexing [45,46]. Moreover, there are very few studies of dermal exposure to fibres or nanowires even though asbestos fibres have been shown to penetrate and persist in skin [47]. 

## 4. Conclusions

The combination of 2D and 3D models for assessing the risk from dermal exposure to nanomaterials is expected to provide an increasingly important methodology as NW are incorporated into consumer products. Our results clearly show that keratinocytes, non-immune cell in the epidermis, rapidly and massively internalized AgNW leading to dose-dependent toxicity. In contrast, our study also demonstrates that the epidermis can provide an effective barrier for NW passage with no identified translocation or irritation end points even after a long exposure time (42 h). The present study, however, represents the first step in full risk assessment for acute dermal exposure to NW on intact epidermis and further studies of NW exposure using models for compromised skin and longer timescales with repeated exposure at low doses that are more realistic will provide a timely assessment of dermal exposure routes. 

## Figures and Tables

**Table 1:** Cell viability determined by MTT assay of reconstructed human epidermis after topical application for 42 h of short, long silver nanowire (AgNWs) and AgNO3 at 600 µg/mL and 3% Sodium dodecyl sulfate (SDS) as positive control of toxicity. Results are expressed as percentage of viability relative to the non-treated control samples as mean ± SD for *n* = 3. * *p* < 0.05 vs. control.

Viability (%)	Water	S-AgNW	L-AgNW	AgNO3	SDS
Mean ± SD	100 ± 2.24	96.63 ± 1.48 *	103.02 ± 4.82	91.88 ± 4.24 *	0.67 ± 0.04 *

## Supplementary Materials

The following are available online at http://www.mdpi.com/2079-4991/8/4/232/s1. Electronic supplementary material (additional figures) as described in the text. Figure S1: SEM images showing the incomplete internalization of AgNW in human primary keratinocytes, Figure S2: SEM images of control non-treated primary keratinocytes, Figure S3: SEM images of cross-section of control non-treated primary keratinocytes, Table S1: Summary of the characteristics of two types of AgNW used in this study: short and long AgNW abbreviated S-AgNW and L-AgNW, Movie S1: 3D reconstruction of confocal images of primary human keratinocytes exposed to long AgNW for 24 h. Images were acquired in different mode: reflectance; transmittance; fluorescence in order to visualize respectively the NW; the cell and the nucleus that was stained with Hoechst. Scale bar represent 5 µm, Movie S2: Time-lapse confocal imaging of the kinetics of uptake of long AgNWs by primary human keratinocytes. Images were acquired each 2 min in reflected and transmitted light mode in order to visualize respectively the NW and the cells.

## Abbreviations

AgNW	silver nanowire
S-AgNW	short AgNW
L-AgNW	long AgNW
AgNP	silver nanoparticle
AgNR	silver nanorod
HPK	human primary keratinocyte
ITO MTT	indium tin oxide 3-[4,5-dimethylthiazol-2-yl]-2,5-diphenyl tetrazolium bromide
RHE	reconstructed human epidermis
SE	secondary-electron
BSE	backscattered-electron
