# Supplementary material for: In Vitro Dermal Safety Assessment of Silver Nanowires after Acute Exposure: Tissue vs. Cell Models
Source: Nanomaterials (Basel). 2018 Apr 11;8(4):232. doi: 10.3390/nano8040232 (PMC5923562; doi:10.3390/nano8040232)
Supplement: Supplementary file 1 [file nanomaterials-08-00232-s001.zip › nanomaterials-281225-SI/nanomaterials-281225-supplementary-final.pdf]

Supplementary Material

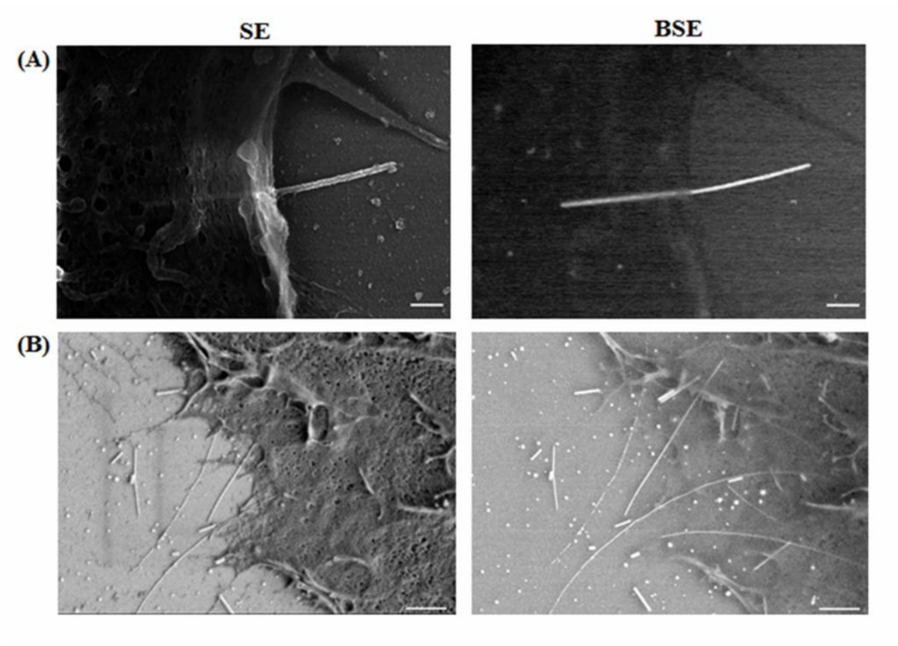

Figure S1. SEM images showing the incomplete internalization of S- (**A**) and L-AgNW (**B**) in primary keratinocytes cells after 24 hours of exposure to 1.5 μg/mL AgNW. Scale bars represent 0.2 μm (**A**) and 1 μm (**B**), respectively. SEM images were acquired using the secondary electron (SE; Left panel) and backscattered electron (BSE; Right panel) detector respectively.

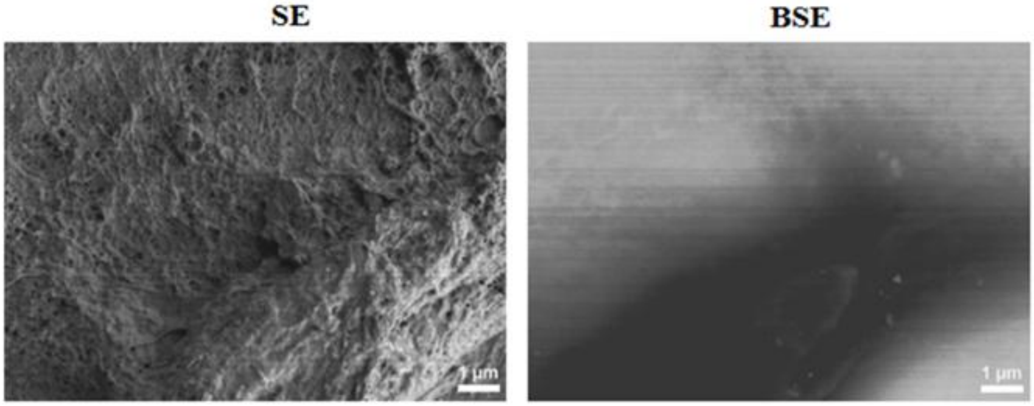

Figure S2. SEM images of non-treated keratinocytes cells. Scale bars represent 1 μm. SEM images were acquired using the secondary electron (SE; Left panel) and backscattered electron (BSE; Right panel) detector respectively.

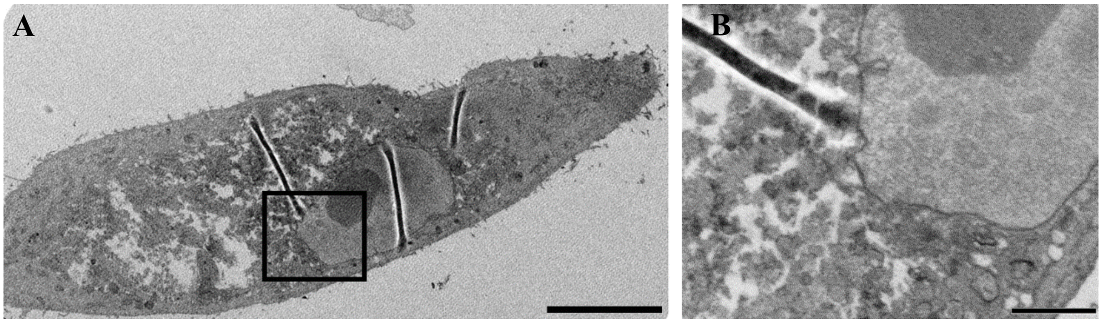

Figure S3. SEM images of cross section of non-treated keratinocytes cell. Image B show a higher magnification of the box area in the image A. Scale bars represent 10 µm (A) and 2 µm (B). SEM images were acquired using the backscattered electron detector.

Table S1. Summary of the characteristics of two types of AgNW used in this study: short and long AgNW abbreviated S-AgNW and L-AgNW. Average diameter and length as well as calculated volume / AgNW, surface area / AgNW and number of AgNW/ ml for the 50µg/ml dilution are indicated.

**Material**	**Diameter (nm)**	**Length (nm)**	**Calculated Volume/AgNW (cm^3)**	**Calculated Surface Area/AgNW (cm^2)**	**Numbers of AgNW/mL in 50 µg/mL Dilution**
S-AgNW	40	2	2.51×10^{-15}	2.53×10^{-9}	1.90×10^{9}
L-AgNW	50	20	3.9×10^{-14}	3.14×10^{-8}	1.21×10^{8}
